# Population susceptibility: A vital consideration in chemical risk evaluation under the Lautenberg Toxic Substances Control Act

**DOI:** 10.1371/journal.pbio.3000372

**Published:** 2019-08-29

**Authors:** Patricia D. Koman, Veena Singla, Juleen Lam, Tracey J. Woodruff

**Affiliations:** 1 Environmental Health Sciences, University of Michigan School of Public Health, Ann Arbor, Michigan, United States of America; 2 Obstetrics, Gynecology and Reproductive Sciences, Program on Reproductive Health and the Environment, University of California San Francisco School of Medicine, San Francisco, California, United States of America; 3 Department of Health Sciences, California State University East Bay, Hayward, California, United States of America; National Institute of Environmental Health Sciences, United States of America

## Abstract

The 2016 Frank Lautenberg Chemical Safety for the 21st Century Act (Lautenberg TSCA) amended the 1976 Toxic Substances Control Act (TSCA) to mandate protection of susceptible and highly exposed populations. Program implementation entails a myriad of choices that can lead to different degrees of public health protections. Well-documented exposures to multiple industrial chemicals occur from air, soil, water, food, and products in our workplaces, schools, and homes. Many hazardous chemicals are associated with or known to cause health risks; for other industrial chemicals, no data exist to confirm their safety because of flaws in 1976 TSCA. Under the 2016 Lautenberg amendments, the United States Environmental Protection Agency (EPA) must evaluate chemicals against risk-based safety standards under enforceable deadlines, with an explicit mandate to identify and assess risks to susceptible and highly exposed populations. Effective public health protection requires EPA to implement the Lautenberg TSCA requirements by incorporating intrinsic and extrinsic factors that affect susceptibility, adequately assessing exposure among vulnerable groups, and accurately identifying highly exposed groups. We recommend key scientific and risk assessment principles to inform health-protective chemical policy such as consideration of aggregate exposures from all pathways and, when data are lacking, the use of health-protective defaults.

## Abstract 2

The Toxic Substances Control Act legally oversees the over 40,000 chemicals in the United States; however, this Perspective article maintains that the US Environmental Protection Agency’s chemical evaluation plans do not reflect current science, recommending methods to improve evaluations to protect public health.

## Introduction

Hazardous industrially manufactured chemicals are ubiquitous in society despite the 1976 Toxic Substances Control Act (TSCA) (Public Law No. 114–182) [1–3]. Hazardous chemicals are found in products such as bedding, furniture, building materials, clothing, cleaning products, food containers, and toys [4,5]. Multiple industrial chemicals are also present in every person in the US, many at levels that can increase the risk of adverse health outcomes [4]. Approximately 9.5 trillion pounds of over 40,000 industrial chemicals are currently in production [3, 6]. Exposures to chemicals such as asbestos, methylene chloride, organic solvents, toxic metals, and halogenated flame retardants can increase the risk of death, cancer, birth defects, and loss of cognitive capacity in children (e.g., see Table 1) [4,7–10]. The costs of environmental chemical exposures are in the billions of US dollars, with one limited study of children estimating $76.6 billion annually (2.7%–4.8% of US healthcare costs) for lead poisoning, asthma, cancer, and developmental disabilities [11,12]. The estimated cost of cleaning up chemical waste at the 294,000 hazardous waste sites across the country is $250 billion, excluding the societal costs of potential health impacts and emerging contaminants [13,14].

**Table 1:** US EPA’s first 10 chemicals for risk evaluation under Lautenberg TSCA, exposures and selected health hazards.

Selected Information From US EPA Scoping and Problem Formulation Documents (based on available information, February 2019)		
Chemical (Other Names or Abbreviations)/CASRN	Uses and Potential Routes of Exposure	Some Identified Health Hazards
1,4-Dioxane^a^/123-91-1	Uses include industrial and commercial processes such as chemical manufacturing and textile processing; present in consumer products (e.g., as a contaminant in shampoo); drinking-water contaminant	Designated “likely to be carcinogenic to humans” (EPA); liver, kidney toxicity
1-Bromopropane (n-propylbromide, 1-BP)^b^/106-94-5	Uses include solvent in industrial and commercial processes such as dry cleaning; consumer products including stain removers; air emissions from industrial facilities	Reproductive/developmental toxicity; neurotoxicity; designated as “reasonably anticipated to be a human carcinogen” (US Department of Health and Human Services, NTP)
Asbestos^c^/1332-21-4	Uses include chemical manufacturing, chlor-alkali industry, brakes; present in wide range of building/infrastructure materials including cement pipes, roofing, flooring, and insulation	Designated as “known to be a human carcinogen” (NTP)
Carbon tetrachloride^d^/56-23-5	Uses include industrial and commercial processes such as chemical manufacturing; water and indoor air contaminant	Designated “likely to be carcinogenic to humans” (EPA); liver, kidney toxicity
Cyclic aliphatic bromide cluster (HBCD)^e^/25637-99-4	Uses include flame retardant in plastics, electronic cases, wire and cables, building insulation, textiles for furniture and floors; indoor air and dust contaminant	Reproductive/developmental toxicity; developmental neurotoxicity; thyroid toxicity
Methylene chloride (Dichloromethane, DCM)^f^/ 75-09-2	Uses include as solvent in industrial and commercial processes for cleaning and degreasing; consumer products including paint strippers and adhesives; air emissions from industrial and commercial facilities; drinking-water contaminant	Designated “likely to be carcinogenic to humans” (EPA); acute toxicity; neurotoxicity
N-Methyl-pyrrolidone (NMP)^g^/ 872-50-4	Uses include as solvent in industrial and commercial processes for cleaning and degreasing; consumer products including paint strippers, adhesives, and printer inks; air emissions from industrial and commercial facilities; drinking-water contaminant	Reproductive/ developmental toxicity; systemic toxicity
Pigment Violet 29 (Anthra[2,1,9-def:6,5,10-d′e′f′]diisoquinoline-1,3,8,10[2H,9H]-tetrone)^h^/ 81-33-4	Uses include in industrial and commercial plastics, rubber, paints, coatings; printing inks; consumer water and acrylic paints	Limited industry-sponsored guideline studies available with sponsors concluding lack of toxicity
Trichloroethylene (TCE)^i^/ 79-01-6	Uses include as solvent in industrial and commercial processes for cleaning and degreasing; consumer products including adhesives, carpet cleaners, and spot removers; air emissions from industrial and commercial facilities; indoor air and drinking-water contaminant	Designated “carcinogenic to humans” (EPA); reproductive/developmental toxicity; neurotoxicity; immunotoxicity
Tetrachloroethylene (perchloroethylene, PERC)^j^ / 127-18-4	Uses include as solvent in industrial and commercial processes for dry cleaning and degreasing; consumer products including adhesives, cleaners, and spot removers; air emissions from industrial and commercial facilities; indoor air and drinking-water contaminant	Designated “likely to be carcinogenic to humans” (EPA); reproductive/developmental toxicity; neurotoxicity

^a^US EPA (2018) Problem Formulation of the Risk Evaluation for 1,4-Dioxane; US EPA (2017) Scope of the Risk Evaluation for 1,4-Dioxane.

^b^US EPA (2018) Problem Formulation of the Risk Evaluation for 1-Bromopropane; US EPA (2017) Scope of the Risk Evaluation for 1-Bromopropane.

^c^US EPA (2018) Problem Formulation of the Risk Evaluation for Asbestos; US EPA (2017) Scope of the Risk Evaluation for Asbestos.

^d^US EPA (2018) Problem Formulation of the Risk Evaluation for Carbon Tetrachloride (Methane, Tetrachloro-); US EPA (2017) Scope of the Risk Evaluation for Carbon Tetrachloride (Methane, Tetrachloro-).

^e^US EPA (2018) Problem Formulation of the Risk Evaluation for Cyclic Aliphatic Bromides Cluster (HBCD); US EPA (2017) Scope of the Risk Evaluation for Cyclic Aliphatic Bromides Cluster.

^f^US EPA (2018) Problem Formulation of the Risk Evaluation for Methylene Chloride (Dichloromethane, DCM); US EPA (2017) Scope of the Risk Evaluation for Methylene Chloride (Dichloromethane, DCM).

^g^US EPA (2018) Problem Formulation of the Risk Evaluation for N-Methylpyrrolidone (2-Pyrrolidinone, 1-Methyl-); US EPA (2017) Scope of the Risk Evaluation for N-Methylpyrrolidone (2-Pyrrolidinone, 1-Methyl-).

^h^US EPA (2018) Problem Formulation of the Risk Evaluation for C.I. Pigment Violet 29 (Anthra[2,1,9-def:6,5,10-d'e'f']diisoquinoline- 1,3,8,10(2H,9H)-tetrone); US EPA (2017) Scope of the Risk Evaluation for C.I. Pigment Violet 29 (Anthra[2,1,9-def:6,5,10-d'e'f']diisoquinoline- 1,3,8,10(2H,9H)-tetrone).

^i^US EPA (2018) Problem Formulation of the Risk Evaluation for Trichloroethylene; US EPA (2017) Scope of the Risk Evaluation for Trichloroethylene.

^j^US EPA (2018) Problem Formulation of the Risk Evaluation for Perchloroethylene (Ethene, 1,1,2,2-Tetrachloro); US EPA (2017) Scope of the Risk Evaluation for Perchloroethylene (Ethene, 1,1,2,2-Tetrachloro).

Abbreviations: CASRN, Chemical Abstracts Services registry number; EPA, US Environmental Protection Agency; Lautenberg TSCA, 2016 Frank Lautenberg Chemical Safety for the 21st Century Act; NTP, National Toxicology Program

Over the past half century, scientists and the public gained a more comprehensive understanding of exposures and health effects from industrial chemicals. Research evaluating exposure to environmental chemicals evolved from directly studying workers to examining consumers and vulnerable populations to assessing potential impacts on future generations (e.g., epigenetics). The understanding of the nature of harm from industrial chemical exposures expanded from one adverse endpoint to many, as well as from one chemical to cumulative exposures and vulnerable periods of exposure across the life course [15]. Health-based regulatory limits have been lowered, not raised, as the science advances [16]. Most importantly, exposures to industrial chemicals and their health consequences remain preventable [17]. Consequently, leading scientists and medical societies have identified environmental pollutants as contributing to adverse health consequences and called for public policies to prevent harmful exposures, emphasizing the need to protect susceptible and highly exposed populations [18–21]. The National Research Council’s report *Science and Decisions*: *Advancing Risk Assessment* recommended improvements to chemical risk assessment to protect public health (referred to hereafter as “*Science and Decisions*”) [15].

In the US, Congress passes laws such as the 1976 Toxic Substance Control Act (TSCA) that mandate the US Environmental Protection Agency (EPA) implement the law through policies, rule makings, and regulations to limit toxic chemical exposures. The authorizing law sets bounds on EPA’s authority, and EPA also has some discretion in implementing the law.

The limitations of 1976 TSCA contributed to a notoriously ineffective implementation that did not protect public health [1–2,22–25]. For example, under 1976 TSCA, EPA did not have adequate authority to require chemical testing prior to chemicals entering commerce [1,2,20–22]. Health and safety testing is available for just 200 chemicals (about 2% of the total manufactured chemicals) [25,26]. Furthermore, EPA could not effectively regulate chemicals with documented adverse health effects, like asbestos and methylene chloride, partly because of the burden of demonstrating “unreasonable risk” along with consideration of the cost to regulate. As a result, hazardous chemicals remained in production and use [27–32]. Faced with mounting evidence of harms and pressure from public health groups, states and other jurisdictions issued their own requirements to fill gaps left by federal inaction. Chemical manufacturers found the variable local requirements to be onerous in a global market, which set the stage for the 2016 Frank Lautenberg Chemical Safety for the 21st Century Act (Lautenberg TSCA) (Public Law No. 114–182) [33].

An important change is that Lautenberg TSCA directs EPA to identify and protect “potentially exposed or susceptible sub-populations,” defined as “a group of individuals within the general population identified by the [US EPA] Administrator who, due to either greater susceptibility or greater exposure, may be at greater risk than the general population of adverse health effects from exposure to a chemical substance or mixture, such as infants, children, pregnant women, workers, or the elderly” (15 USC §2602 [12]) [34]. The law further requires that EPA decisions under Lautenberg TSCA must protect such populations (15 USC §2604 [a][3][A]; 15 USC §2605 [b][1][B][i], [b][4][A], and [h][1][B]) [35–38]. Finally, the amended law articulates scientific standards: “to the extent that the [U.S. EPA] Administrator makes a decision based on science, the Administrator shall use scientific information, technical procedures, measures, methods, protocols, methodologies, or models, employed in a manner consistent with the best available science” (15 USC §2625 [h]) [39]. In this context, we discuss scientific risk assessment principles necessary to meet legal requirements to safeguard the health of susceptible and highly exposed populations under Lautenberg TSCA. These principles are articulated in *Science and Decisions* and other documents and based on current scientific understanding of chemical exposures and biological and health effects [15,40–42] (Table 2). These provisions are consistent with the significant agreement among the public health community that US chemical policy should reflect contemporary science and provide public health protection, especially for susceptible and highly exposed groups [43–45].

**Table 2:** Recommendations for US EPA to support scientifically based health-protective chemical policy considering susceptible and highly exposed populations.

Recommendations for primary prevention Support the strongest protections for human health, especially regarding susceptible and highly exposed populations, in EPA’s interpretation of the legal requirements of Lautenberg TSCA. Environmental exposures to harmful industrial chemicals are a preventable source of adverse health consequences [18,46].	
Vulnerable populations	Identify and assess aggregate exposures to susceptible and highly exposed populations including but not limited to children, pregnant women, elderly, workers (including people planning families), and fenceline communities as required by law (Fig 1) [15,47,48]. Improve the basis of accounting for variability and susceptibility across the population by identifying potential susceptible populations based on established, scientifically supported extrinsic and intrinsic factors that increase vulnerability [41,49].
Aggregate exposure	Account for aggregate exposures—people’s exposures to the same chemical from all uses and through multiple exposure pathways (such as air, water, food, dermal contact), including all pathways that can be reasonably anticipated [15,41,50].
Health-protective defaults	Given limited data for a particular chemical or exposure, when necessary data cannot be developed in a timely way, use evidence-based health-protective defaults that reflect the range of variability and susceptibility in the population to ensure risks are not underestimated (e.g., child-specific defaults, pregnancy defaults) [15,42].
Windows of susceptibility	Identify and evaluate timing of “windows of susceptibility” to toxic chemicals during development or other sensitive life stages [51]. Ensure adequate data and/or defaults to assess and address the timing of these impacts.
Cumulative exposure and risk	Account for populations’ simultaneous exposure to a multitude of different chemicals and social stressors in the real world, many of which contribute to similar adverse health effects resulting in increased risk (cumulative risks, see Fig 1) [15, 52].
Uncertainty	Appropriately characterize uncertainty by developing and further integrating monitoring, measurement, and modeling efforts and communicating levels of confidence to support decision-making [15]. Ensure sufficient data to characterize factors that influence uncertainty in the risk evaluations.

Abbreviations: EPA, US Environmental Protection Agency; Lautenberg TSCA, 2016 Frank Lautenberg Chemical Safety for the 21st Century Act

Although Lautenberg TSCA introduced some potential improvements, implementation of the law leaves critical decisions to EPA [45]. Lautenberg TSCA requires that EPA determine to what extent chemicals pose an “unreasonable risk” to health based solely on scientific data and irrespective of compliance costs. It also requires that EPA ensures chemical uses do not pose an “unreasonable risk” to susceptible and highly exposed populations, such as pregnant women, children, workers, and the elderly. However, the amended law does not require chemical manufacturers to provide a minimum set of data on health risks and exposure for susceptible and highly exposed groups. Furthermore, Lautenberg TSCA did not fully define unreasonable risk, and EPA must develop an operational definition as well as its specific risk evaluation and decision-making processes. Thus, EPA must determine the details of how to collect and assess scientific evidence for determining risks and what information to require from manufacturers or its own research to meet statutory requirements. In implementing Lautenberg TSCA, EPA will set precedents for the type of scientific data necessary to collect and the assessment of susceptible populations and exposures across its current and future risk evaluation decisions. This process is occurring primarily through two steps: (1) general provisions in the final “framework rules” (Risk Prioritization: July 20, 2017 [FR 33753][FRL–9964–24], Risk Evaluation: July 20, 2017 [FR 33726][FRL–9964–38]) [53,54] and (2) each specific chemical risk evaluation.

Public health protection will be heavily dependent on these federal decisions because Lautenberg TSCA includes new state preemption provisions—meaning that states are precluded from taking further action once EPA determines that a chemical does not pose an unreasonable risk or when EPA takes final action in the risk management phase (Public Law No. 114–182) [33]. New state action is paused during EPA’s risk evaluation of high-priority chemicals.

In Table 2, we recommend scientific principles EPA should incorporate to assure adequate assessments of susceptible and highly exposed populations to support health-protective chemical policy as required by law. In the next sections, we analyze EPA decisions to date (as of June 2019) with a focus on susceptibility and exposure considerations that are now required under Lautenberg TSCA. We acknowledge there are many other factors contributing to risk, including cumulative impacts, timing of exposures during sensitive periods of human development, and uncertainty in the data (see Table 2).

### Population susceptibility

**Fig 1:**
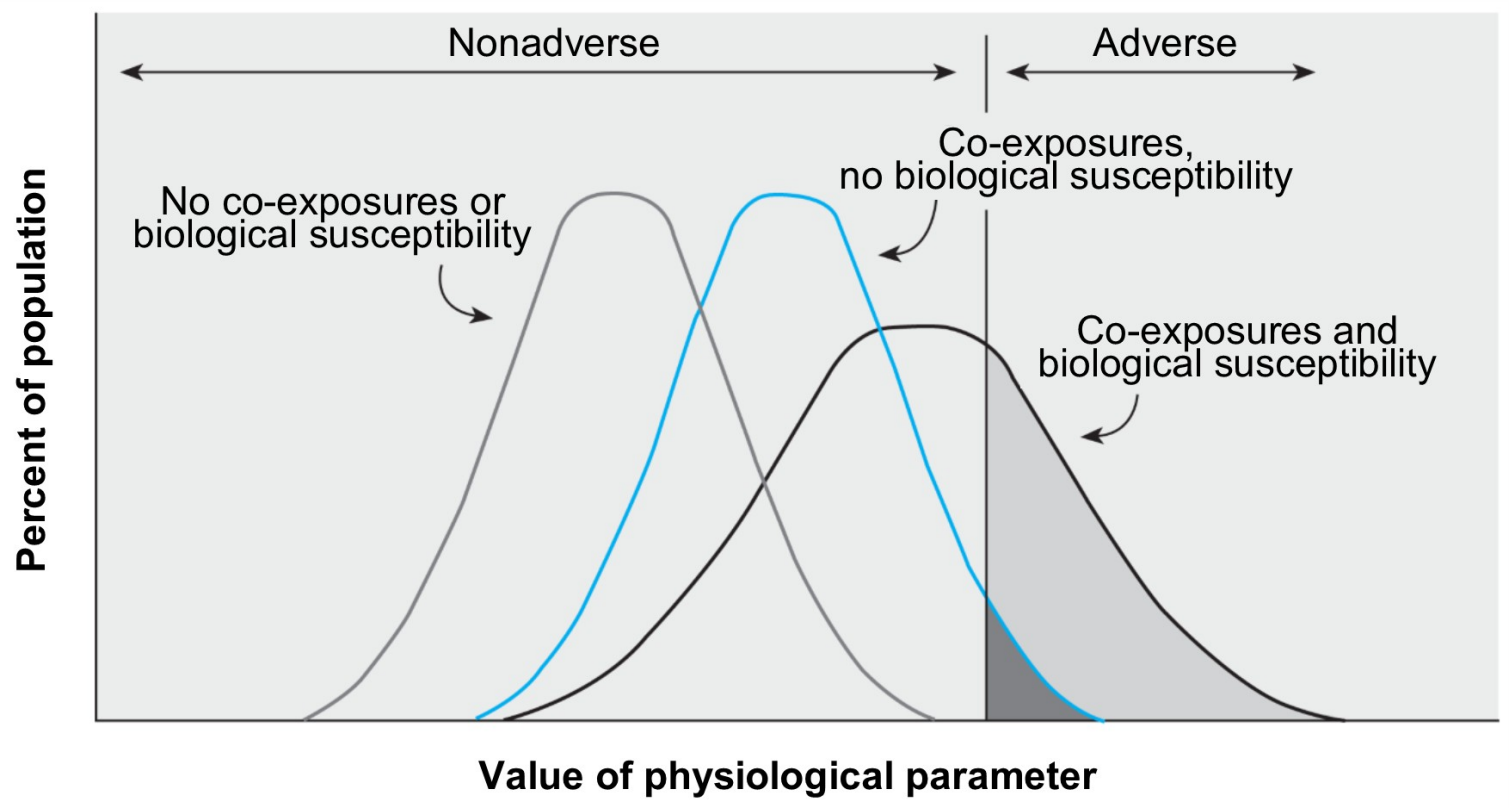
How coexposures to other chemical stressors and variability in biological susceptibility combine to influence population risk. *Figure reproduced with permission from* Environmental Health Perspectives [55].

As shown in Fig 1, population variability in susceptibility and coexposures combine to determine biological response to chemical exposure [55]. To accurately identify subpopulations at greater risk, EPA’s analysis must incorporate the current scientific understanding of factors that contribute to greater susceptibility and to greater or more impactful exposures. These include intrinsic factors (e.g., life stage, genetics, underlying disease status, nutrition), extrinsic factors (e.g., social and life circumstances such as poverty and life stress), and exposures to other chemicals (Fig 1) [15,56].

Unfortunately, in its assessment plans for the first 10 chemicals (as of June 2019), EPA has not yet incorporated these established, scientifically supported intrinsic and extrinsic factors that increase susceptibility or exposure [57]. For eight of the first 10 evaluations (i.e., perchloroethylene; asbestos; trichloroethylene; N-Methyl-pyrrolidone [NMP]; methylene chloride; carbon tetrachloride; 1,4-dioxane; and pigment violet 29), EPA does not currently identify pregnant women, infants, children, families living near current and former industrial sites, or any other potentially highly exposed or susceptible subpopulation under the amended TSCA (as of June 2019) [58–66]. For example, the prenatal life stage can be the most sensitive to developmental and reproductive toxicants [8,21], and people of child-bearing age are a susceptible subpopulation for chemicals with such hazards [67], such as trichloroethylene. EPA’s Integrated Risk Information System (IRIS) assessment of trichloroethylene concluded that toxicity to the developing fetus was one of the most sensitive observed adverse effects [68]; however, EPA’s TSCA risk evaluation omits this consideration.

For many industrial chemicals, there is ample evidence from the literature and IRIS assessments of increased susceptibility due to age, life stage, preexisting disease, genetic variation, and many other factors that should be incorporated into the TSCA evaluations [29–31,68–71]. In general, populations with these and other established factors should be considered a susceptible population for each chemical, unless there are chemical-specific data showing otherwise.

*Science and Decisions* recommends that risk assessments should quantitatively incorporate factors like susceptibility that influence the likelihood of disease and, when specific data are lacking, incorporate scientifically based default values in their assessments [15,49]. For example, the California EPA developed risk values for chemicals (e.g., atrazine, chlorpyrifos, lead, nickel, manganese) that address child-specific routes of exposure and differences in children’s susceptibility compared to adults [72]. For cancer, *Science and Decisions* recommended 25 as a reasonable default value to include in the calculation of risk to account for the population variability in response to chemical exposure between the median individual and those with more extreme responses [15]. Because the scientific basis for these defaults is already developed, EPA could easily integrate them into assessments. If EPA fails to incorporate established science to adequately identify and assess susceptible and highly exposed groups, the resulting risk determinations will underestimate risk of a chemical and fail to protect public health, as required by law.

### Highly exposed populations

Established scientific principles for exposure assessment, including from EPA guidance documents, recognize the importance of including aggregate exposures to accurately detect highly exposed populations [43,50,56]. Aggregate exposure is defined as the combined exposures to an individual from a single chemical substance from all uses and across multiple pathways (such as air, water, food, dermal contact). However, EPA’s statements in the first 10 chemical problem formulations and framework rules indicate that it will not conduct full aggregate assessments; instead, EPA plans to consider exposure pathways in isolation and will separate and narrow which chemical uses will be included (called “conditions of use”) (see Table 1 references). These decisions systematically underestimate risk. Specifically, this approach could miss populations with greater exposures by excluding contemporary exposures from past common chemical uses (e.g., asbestos in buildings and flame-retardant chemicals in furniture, textiles, and electronics); reasonably foreseeable ongoing chemical uses contaminating land, air, and water; and uses for which a chemical is present unintentionally as a contaminant or by-product.

For instance, regarding previously common uses of the flame-retardant cyclic aliphatic bromide cluster (hexabromocyclododecane, HBCD), EPA states, “There is no longer manufacture, processing or distribution of HBCD for [high-impact polystyrene] HIPS or textiles; and therefore, those uses are not included in the scope of the risk evaluation of HBCD” [62]. HBCD was used as an additive flame retardant in HIPS casing for electronics such as TVs, DVD players, and computers. The number of home electronics has been correlated with the amount of HBCD on people’s hands (an exposure metric used to estimate dermal absorption and hand-to-mouth ingestion), indicating that flame retardant use in home electronics is a significant current source of exposure for the general population [73]. An exposure calculation excluding this ongoing source would underestimate exposure to HBCD. Furthermore, because of increased hand-to-mouth activities, toddlers and young children, a potential susceptible subpopulation, can have greater exposures to environmental chemicals compared to adults because of their behaviors and physiological differences [48,74–77]. EPA’s draft risk evaluations systematically exclude previously common uses of chemicals (which EPA calls “legacy” uses) (see [62] and EPA Risk Evaluation final rule: July 20, 2017 [FR 33726][FRL–9964–38] [54]) despite ongoing exposures.

Accurate assessment of aggregate exposure is important because it can reveal risks to susceptible populations that would be missed if only a single exposure source was considered. This is illustrated by EPA’s 2005 risk assessment of the pesticide sulfuryl fluoride (67 FR 5740, February 7, 2002, as amended at 69 FR 3257, January 23, 2004; 70 FR 40908, July 15, 2005, Title 40, Chapter I subpart E, Part 180 Subpart C, Sect. 180.575) [78–80]. With an aggregate exposure assessment, EPA concluded that most people in the US are not exposed to unsafe levels of fluoride, yet “aggregate fluoride exposure for infants and children under the age of 7 years old, where drinking water contains high levels of natural fluoride, exceeds the level that can cause severe dental fluorosis” [81]. Had EPA only considered the risk from fluoride residues contributed by sulfuryl fluoride in isolation, its assessment would not have identified the existing risks to infants and children, a susceptible population (76 *Federal Register* 3421 [January 19, 2011]) [82]. Thus, aggregate exposure assessment of all sources and pathways is critical for EPA to accurately identify the populations most at risk. Note that although pesticides are excluded from TSCA, this example demonstrates an appropriate evaluation of aggregate exposure that can be applied to any chemical.

## Conclusions

Much is at stake for the public’s health and the role of science in decision-making with Lautenberg TSCA implementation. EPA’s decisions over the next several years will influence the level of toxic chemicals in our homes, communities, and bodies. Exposures to industrial chemicals and their harmful health consequences are preventable. If current levels of exposure to chemicals continue unabated, the consequences will be an even greater toxic legacy for future generations, especially for susceptible and highly exposed populations. For eight of the first 10 risk evaluations (as of June 2019), EPA does not identify pregnant women, infants, children, families living near current and former industrial sites, or any other potentially highly exposed or susceptible subpopulation. For all regulated chemicals, EPA must act quickly to identify susceptible and highly exposed populations, evaluate risks, and safeguard health through primary prevention. The challenge ahead for EPA is to incorporate current scientific principles and address the data deficits in the process of identifying, evaluating, and mitigating unreasonable risks. By adopting these recommendations regarding susceptibility and exposure, EPA will ensure that it is accounting for risks to the whole population and thus set the stage for risk management that yields widespread public health benefits.

## Abbreviations

CASRN: Chemical Abstracts Services registry number
EPA: United States Environmental Protection Agency
HBCD: hexabromocyclododecane
HIPS: high-impact polystyrene
IRIS: Integrated Risk Information System
Lautenberg TSCA: 2016 Frank Lautenberg Chemical Safety for the 21st Century Act
NMP: N-Methyl-pyrrolidone
NTP: National Toxicology Program
TSCA: 1976 Toxic Substances Control Act
